# Concomitant BET and MAPK blockade for effective treatment of ovarian cancer

**DOI:** 10.18632/oncotarget.6309

**Published:** 2015-11-12

**Authors:** Ying Jing, Zhenfeng Zhang, Pengfei Ma, Shimin An, Ying Shen, Liang Zhu, Guanglei Zhuang

**Affiliations:** ^1^ State Key Laboratory of Oncogenes and Related Genes, Renji-Med X Clinical Stem Cell Research Center, RenJi Hospital, School of Medicine, Shanghai Jiao Tong University, Shanghai, China; ^2^ State Key Laboratory of Oncogenes and Related Genes, Shanghai Cancer Institute, RenJi Hospital, School of Medicine, Shanghai Jiao Tong University, Shanghai, China; ^3^ Department of Pharmacology and Chemical Biology, Shanghai Jiao Tong University School of Medicine, Shanghai, China; ^4^ Shanghai Collaborative Innovation Center for Translational Medicine, Shanghai, China; ^5^ Shanghai Key Laboratory of Gynecologic Oncology, Ren Ji Hospital, School of Medicine, Shanghai Jiao Tong University, Shanghai, China

**Keywords:** ovarian cancer, BET inhibitors, MEK inhibitors, apoptosis, targeted therapy

## Abstract

Ovarian cancer is the most lethal gynecologic malignancy, and it is imperative to develop new treatments to ameliorate patient survival. Using an anti-cancer drug library containing 180 small molecule inhibitors, we performed a high-content image-based screen and found that BET and MEK inhibitors are among the candidates which were able to effectively inhibit ovarian cancer cell growth. However, BET inhibition alone was largely cytostatic, possibly due to feedback activation of the MAPK pathway. Consequently, the combination of MEK and BET inhibitors suppressed both cell proliferation and survival, and was more efficacious than single agent. Mechanistically, BET and MEK inhibitors exerted synergistic effects on apoptosis regulators including BIM and BAD. Our findings support concomitant BET and MAPK blockade as an effective therapeutic strategy in ovarian cancer.

## INTRODUCTION

Ovarian cancer contributes to the highest mortality among gynecological cancers [1–3]. Current standard treatment is debulking surgery followed by taxane-platinum chemotherapy [4]. Despite good initial responses, almost all patients have disease recurrence or progression [5]. As a result, the slope of survival improvement achieved with conventional treatment has reached a plateau, and novel targeted therapeutics are urgently needed to improve patient outcome [6, 7].

Recently, bromodomain and extraterminal (BET) domain protein BRD4 has been identified as a potential therapeutic target in ovarian cancer [8]. The BET family proteins function as epigenetic “readers” that bind the acetylated lysine residues on histone tails [9–11], and play a critical role in the acetylation-dependent assembly of transcriptional regulator complexes governing the expression of key oncogenes and anti-apoptotic genes [12, 13]. Suppression of BRD4 using specific BET bromodomain inhibitors JQ1 or I-BET151 led to robust and broad antitumor effects across various subtypes of ovarian cancer. However, we discovered that BET inhibitors mainly induce cell-cycle arrest in ovarian cancer instead of cell apoptosis, which may limit their clinical usage in patients with advanced ovarian cancer. In this study, we sought to identify better drug regimens or combinations as more effective therapeutic approaches in ovarian cancer.

We synthesized an anti-cancer drug library composed of both experimental compounds and early or advanced stage clinical candidates, and performed a high-content image-based screen in multiple ovarian cancer cell lines. In addition to JQ1, several compounds, including two independent MEK inhibitors, elicited greater than 50% reduction in tumor cell growth with half maximal inhibitory concentration (IC50) of less than 500 nM. Interestingly, we found that BET bromodomain inhibitors relieved feedback inhibition of the MAPK pathway and thus caused hyperactivation of MAPK and a robust upregulation in downstream signaling. This feedback activation was suppressed and tumor cell inhibition was enhanced with combined administration of BET and MEK inhibitors, which may represent a novel therapeutic strategy in ovarian cancer.

## RESULTS

### BET bromodomain inhibition resulted in cell growth arrest in ovarian cancer

**Figure 1:**
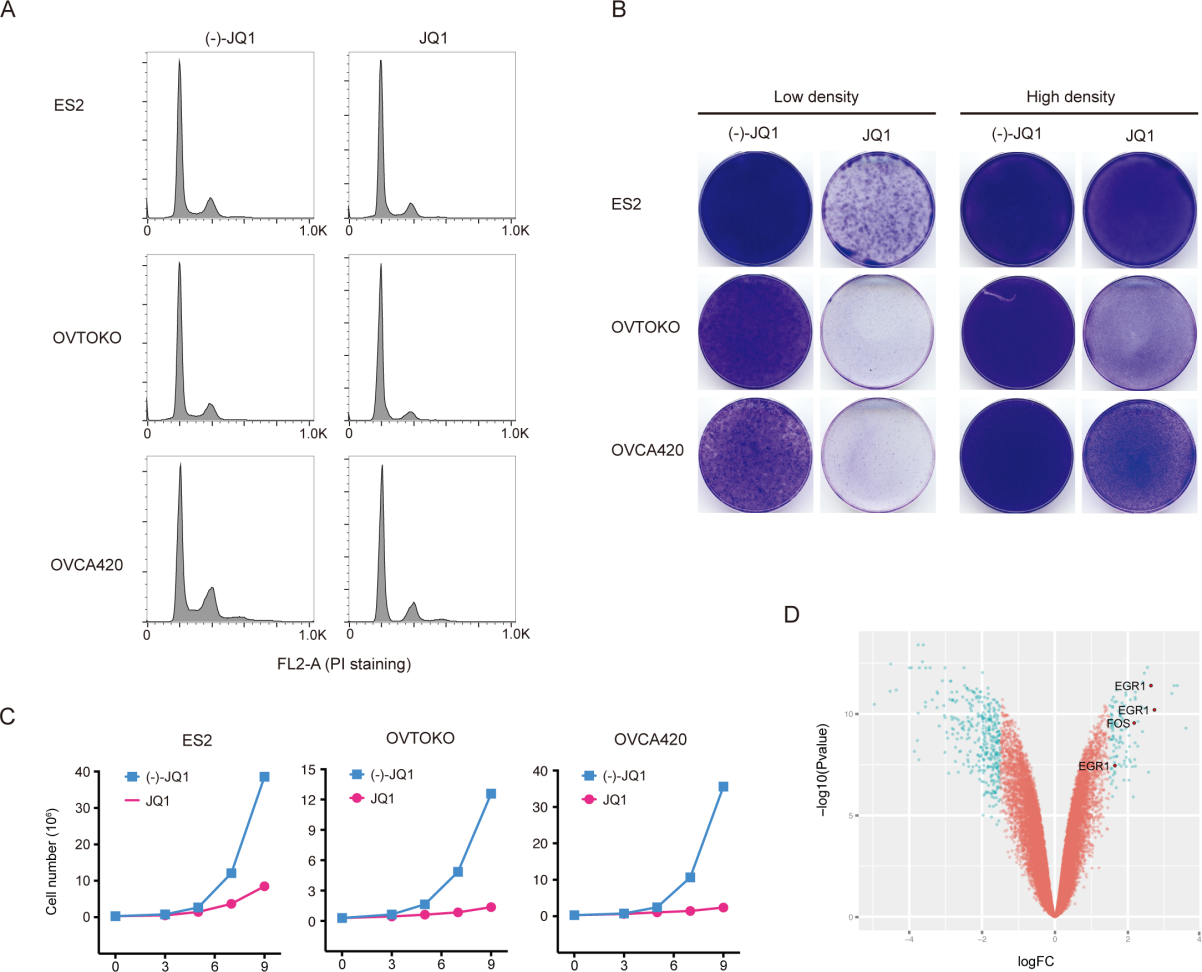
BET bromodomain inhibition resulted in cell growth arrest in ovarian cancer **A.** Cell cycle analysis following 24-hour treatment with (−)-JQ1 or JQ1 (1 μM). **B.** Ovarian cancer cells were seeded at low or high density and treated with JQ1 (1 μM) for two weeks. Cells were fixed with methanol and stained with crystal violet. Low density, 2500 cells per well; High density, 50000 cells per well. **C.** Growth curves of ovarian cancer cell lines treated with JQ1 (1 μM). **D.** Volcano plot of differentially expressed genes in OVTOKO cells treated with JQ1 (1 μM) compared to control.

BET bromodomain inhibitor JQ1 exhibited broad anti-proliferative effects in a large panel of ovarian cancer cell lines (Supplementary Table 1 and unpublished data), without noticeable discrepancy between histological or molecular subtypes (Supplementary Figure 1). We found that JQ1 profoundly arrested cell-cycle without inducing apparent apoptosis in the majority of ovarian cancer cell lines (Figure 1A). To elaborate on this point, we grew three ovarian cancer cell lines (ES2, OVTOKO and OVCA420) at low or high density and treated them with JQ1. At low cell density, JQ1 exhibited a dramatic inhibition on tumor cell growth, while the effect was relatively modest at high cell density (Figure 1B). We further quantified cell growth in the presence of JQ1 at different time points and found that JQ1 was largely a cytostatic drug displaying anti-proliferative but not pro-apoptotic effects on ovarian cancer cells. Indeed, although cell viability was significantly inhibited by JQ1 treatment compared to negative control, there was still a gradual increase of total cell numbers across three ovarian cancer cell lines, indicating that JQ1 did not lead to massive cell death and cancer regression (Figure 1C). Interestingly, microarray analysis showed that gene expression of EGR1 and FOS, two putative targets downstream of the MAPK pathway [14], were elevated in JQ1 treated OVTOKO cells (Figure 1D), suggesting that MAPK might be activated and played a role in JQ1 therapy.

### High-throughput drug screen identified BET and MEK inhibitors as effective drug combination for ovarian cancer

**Figure 2:**
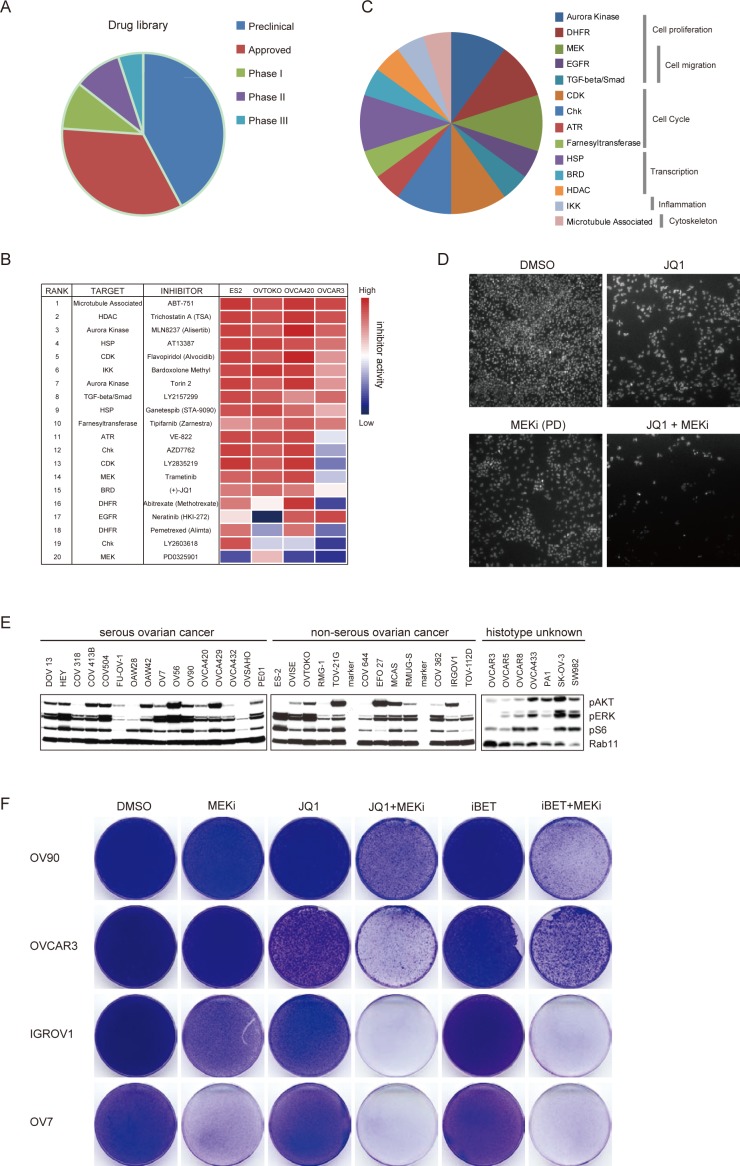
High-throughput drug screen identified BET and MEK inhibitors as effective drug combinations for ovarian cancer **A.** Pie charts depicted clinical status of 180 inhibitors in this study. **B.** Top 20 ranked compounds suppressing ovarian cancer cell growth in the presence of JQ1 and corresponding targets were presented in an inhibitory activity heatmap format. **C.** Distribution of top 20 drugs targeting distinct cellular biological pathways. **D.** Representative images of cells exposed to indicated inhibitors and counter stained by DAPI. PD: PD325901. **E.** Western blot analysis of pAKT, pERK, pS6 and Rab11 in a panel of ovarian cancer cell lines. **F.** Cells were treated with indicated compounds (1 μM) for two weeks, fixed with methanol and stained with crystal violet. MEKi: Trametinib.

To discover effective therapeutic strategies for ovarian cancer, we performed an unbiased image-based high-throughput screen in ovarian cancer cell lines against a library of 180 anti-tumor therapeutics spanning multiple stages of clinical development (Supplementary Table 2). This drug panel is composed of both chemotherapeutic medications and targeted agents directed against a range of key regulators of cell proliferation and survival, including oncogenic kinases, hormone receptors, apoptosis regulators, transcription machinery and DNA damage sensors. More than 30% of these drugs are FDA-approved compounds and 25% have already been in clinical trials (Figure 2A). Considering ovarian cancer heterogeneity, four cell lines derived from serous or non-serous ovarian carcinoma were included in the screen. Using an image-based cell viability assay, small-molecule drugs were initially screened at the same concentration (500 nM) on the ovarian cancer cells. Forty-one compounds induced more than 50% reduction of cell density in all four cell lines and were triaged for further inspection. To specifically identify effective drug combinations with BET bromodomain inhibitors, we further screened each drug in the presence or absence of a fixed dosage of JQ1 in OVCA420 cells. Twenty compounds resulted in a synergistic inhibition on cell growth with JQ1 (Figure 2B). The targets of top-ranked inhibitors spanned multiple crucial cellular processes, such as cell proliferation (Torin 2, Abitrexate, Pemetrexed, PD0325901, Trametinib and Neratinib), cell cycle (Flavopiridol, LY2835219, AZD7762, LY2603618, VE-822 and Tipifarnib), and gene transcription (AT13387, Ganetespib, JQ1 and Trichostatin A) (Figure 2C). Notably, two MEK inhibitors (PD0325901 and Trametinib) were effective in eliminating ovarian cancer cells in combination with JQ1 (Figure 2D), consistent with our observation that JQ1 treatment activated MAPK targets EGR1 and FOS. Furthermore, we assayed a large panel of serous and non-serous ovarian cancer cell lines and found phospho-ERK strongly expressed in most cell lines, indicating that the MAPK pathway was broadly active in ovarian cancer (Figure 2E). Finally, we tested both JQ1 and I-BET151 in combination with Trametinib in the colony formation assay and confirmed the synergism between BET and MEK inhibitors (Figure 2F). Thus, the unbiased pharmacologic interrogation of ovarian cancer cells unequivocally identified BET and MEK inhibitors as effective drug combinations for ovarian cancer.

### BET bromodomain inhibitors activated the MAPK pathway in ovarian cancer

**Figure 3:**
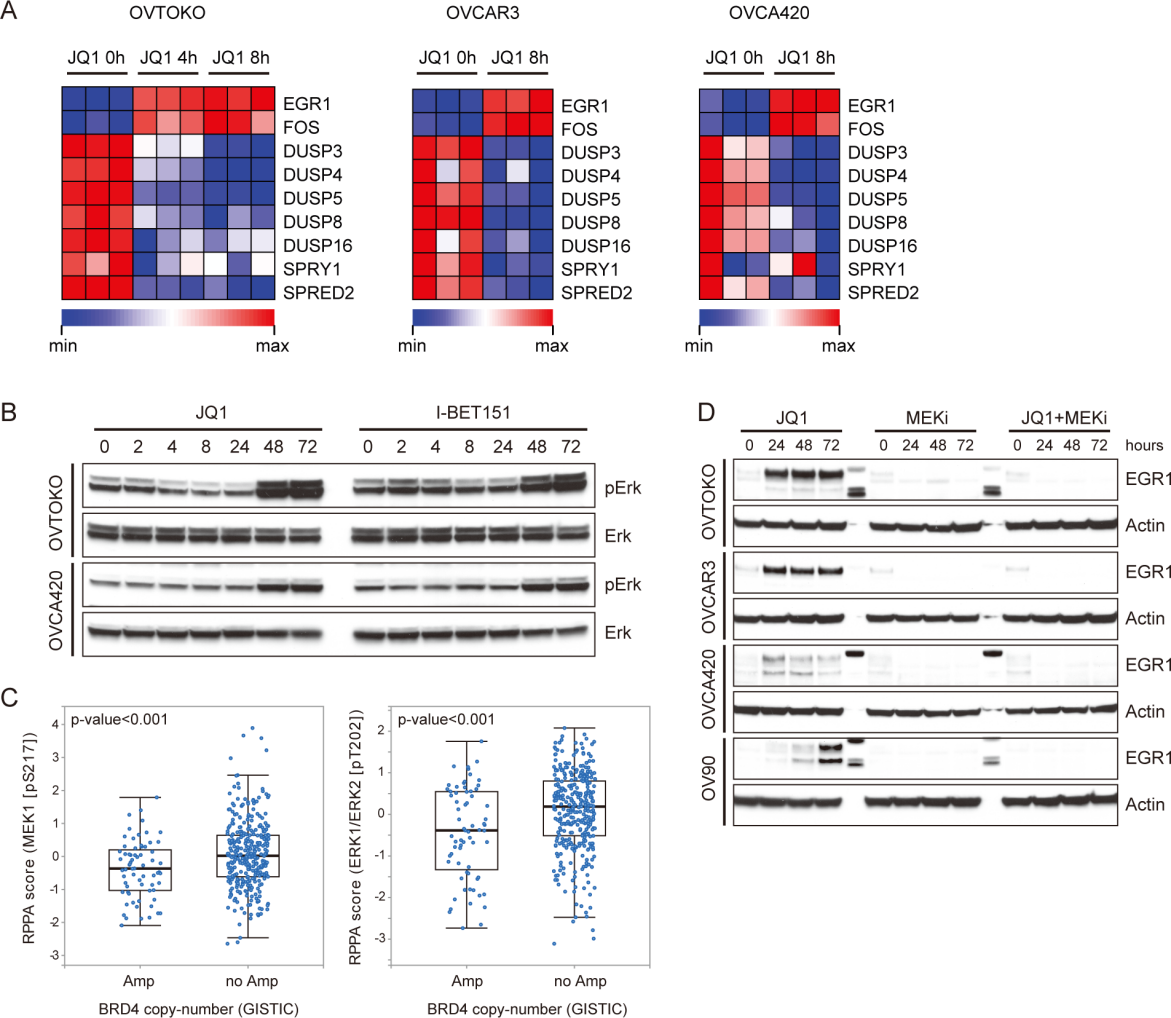
BET bromodomain inhibitors activated the MAPK pathway in ovarian cancer **A.** Heatmap of MAPK pathway molecules expression in JQ1 (1 μM) treated ovarian cancer cells at indicated time point. **B.** Western blot analysis of pERK and ERK in cells treated with JQ1 or I-BET151. **C.** Analysis of MEK1 and ERK1/2 phosphorylation in TCGA ovarian carcinomas with or without BRD4 copy number amplification. **D.** Western blot analysis of EGR1 and actin in ovarian cancer cells exposed to specified inhibitors (1 μM). MEKi: Trametinib.

The synergistic effect of BET and MEK inhibitors prompted us to further decipher the crosstalk between BET proteins and the MAPK pathway in ovarian cancer. As illustrated above, microarray analysis in OVTOKO cells revealed that gene expression of EGR1 and FOS were increased upon JQ1 treatment. Induction of these MAPK targets correlated with decreasing levels of Sprouty1, Spred2 and the ERK phosphatase DUSP proteins, all of which were negative regulators of MAPK signaling cascade [15–17] (Figure 3A). Similar results were achieved in OVCAR3 and OVCA420 cells (Figure 3A), implying that JQ1 activated the MAPK pathway perhaps by release of a negative regulatory signal normally coordinating BET and MAPK activities. Indeed, in ovarian cancer cells treated with JQ1 or I-BET151 inhibitors, we observed a pronounced upregulation in pERK (Figure 3B). These data suggested a model in which BET proteins suppressed MAPK activity. Consistently with this model, analysis of reverse phase protein arrays (RPPA) in the cancer genome atlas (TCGA) database revealed that ovarian carcinomas with BRD4 amplification showed significantly downregulated pMEK and pERK compared with those without BRD4 amplification (Figure 3C; Supplementary Table 3). Importantly, JQ1-induced feedback activation of MAPK signaling as measured by EGR1 expression was completely abrogated by the co-treatment with MEK inhibitor Trametinib (Figure 3D).

### BET and MEK inhibitors synergistically elicited tumor cell apoptosis

**Figure 4:**
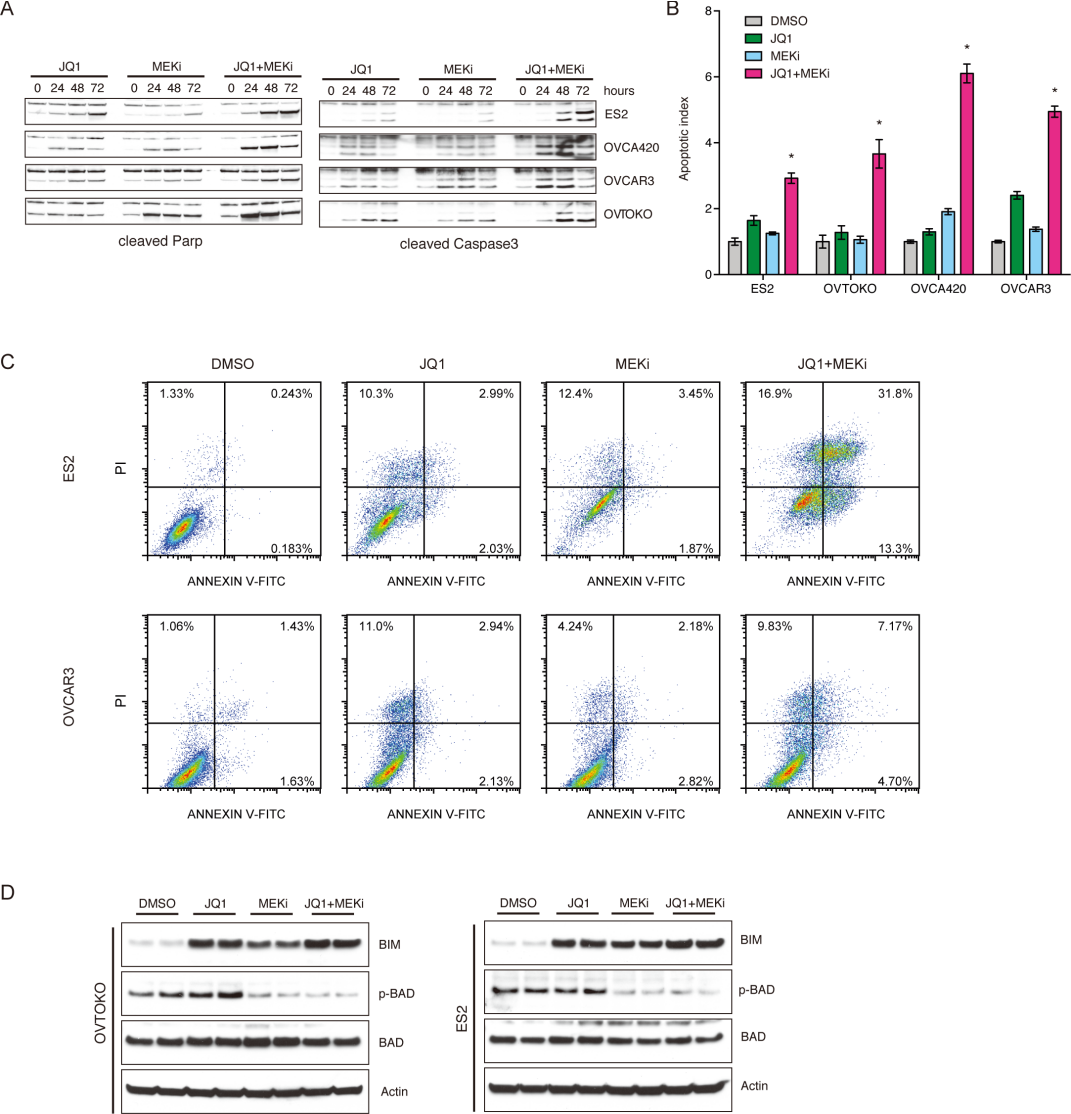
BET and MEK inhibitors synergistically elicited tumor cell apoptosis **A.** Ovarian cancer cells were respectively treated with JQ1, MEKi (Trametinib), and JQ1+MEKi (Trametinib). Cleaved PARP and cleaved Caspase3 expression at different time points were determined by Western blot. **B.** Cell apoptosis induced by indicated inhibitors was quantified using Caspase-Glo3/7 assays. *P < 0.05, ANOVA followed by Tukey tests. **C.** Cell apoptosis analysis following 6 days of treatment with DMSO, JQ1, MEKi (Trametinib), and JQ1+MEKi (Trametinib). Cells were dyed with Annexin V-FITC/PI. **D.** Western blot analysis of BIM, phospho-BAD, BAD and actin in ovarian cancer cells exposed to DMSO, JQ1, MEKi (Trametinib), and JQ1+MEKi (Trametinib). Concentration of the inhibitors was 1 μM.

The molecular mechanisms underlying the synergistic effects of BET and MAPK inhibitors were investigated in ovarian cancer. We reasoned that besides the cytostatic effects of BET inhibitors as single agent, the combination therapy might additionally promote cell apoptosis. Exposure to JQ1 and Trametinib dramatically accelerated apoptosisas indicated by cleaved PARP [18] and cleaved Caspase 3 [19], compared to individual treatment (Figure 4A). Substantial induction of apoptosis was observed in all four cell lines treated with JQ1 and Trametinib, as assessed by both caspase 3/7 activity (Figure 4B) and Annexin V cytometry (Figure 4C; Supplementary Figure 2). Mechanistically, concurrent JQ1 and Trametinib treatment induced upregulation of pro-apoptotic BIM [20] and reduction of anti-apoptotic phospho-BAD [21] (Figure 4D). Taken together, we concluded that BET and MEK inhibitors synergistically elicited tumor cell apoptosis by coordinately regulating apoptosis molecules including BIM and BAD.

### Combined treatment with BET and MEK inhibitors suppressed ovarian tumor growth *in vivo*

**Figure 5:**
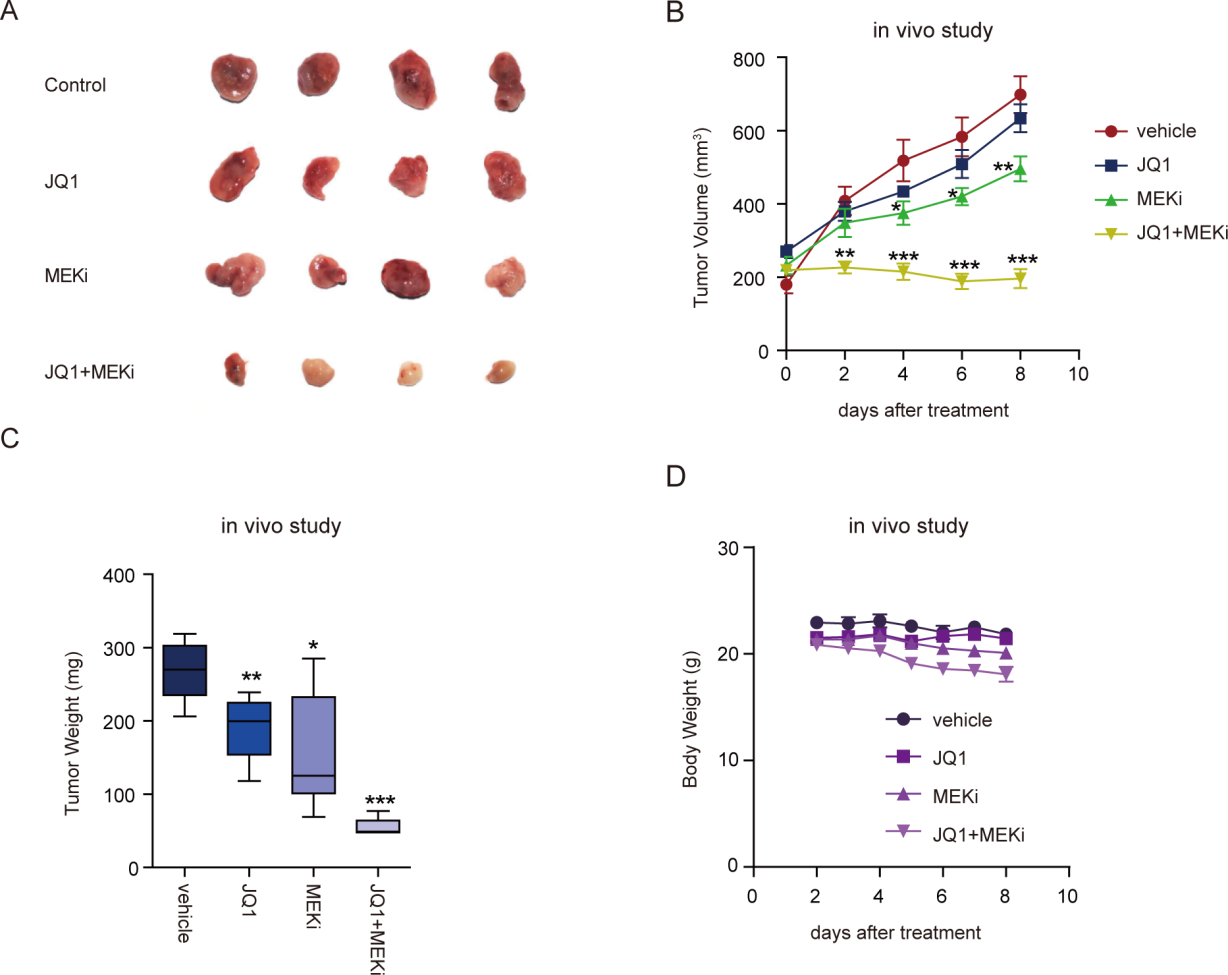
Combined treatment with BET and MEK inhibitors suppressed ovarian tumor growth *in vivo* **A.** Images of ES-2 tumors after eight days of treatment with indicated inhibitors. **B.** Growth curves of ES-2 tumors treated with the indicated drugs. Tumor volume was measured every two days (six mice per group). *P < 0.05; **P < 0.01; ***P < 0.001, unpaired Student's *t*-test. **C.** Tumor weights of ES-2 tumors treated with the indicated drugs for 8 days (six mice per group). *P < 0.05; **P < 0.01; ***P < 0.001, unpaired Student's *t*-test. **D.** Body weights of nude mice bearing ES-2 tumors treated with the indicated drugs were measured everyday (six mice per group).

To explore the *in vivo* anti-tumor effect of BET and MEK inhibitors in ovarian cancer, we subcutaneously transplanted ES2 cells into nude mice. We started to treat nude mice with indicated drugs when the tumor volume reached about 200 mm^3^. After eight days of treatment, we observed significant decrease of tumor volume and tumor weight in xenografts treated with JQ1 and Trametinib polytherapy, compared with vehicle or either drug alone (Figure 5A–5C). Mice weights were monitored to evaluate the possible overt systemic toxicity of combination therapy. Notably, a moderate but significant weight loss was observed upon multiple doses of dual treatment (Figure 5D), suggesting that toxicity might be a dose-limiting factor and needs to be thoroughly investigated before testing the regimens in patients. Nevertheless, concomitant BET and MAPK blockade was generally tolerable and highly effective as a potential therapeutic strategy of ovarian cancer.

## DISCUSSION

We and others have discovered BET bromodomain inhibition as a new treatment approach against ovarian cancer. However, BET bromodomain inhibitors mainly induce cell-cycle arrest, but not apoptosis in ovarian cancer cells, which is likely a potential obstacle of their application in treating ovarian cancer patients. In this study, we identified previously undescribed BET and MEK inhibitor combination as an effective therapeutic strategy in ovarian cancer using an unbiased small-molecule screen platform. BET and MEK inhibitors not only arrested cell growth but also synergistically elicited tumor cell apoptosis, presumably by coordinately regulating apoptosis proteins such as BIM and BAD. Hence, we addressed a high unmet therapeutic need for the treatment of ovarian cancer. It is noteworthy that our chemical screen also uncovered other inhibitors as promising drug candidates to be combined with BET bromodomain inhibitors. These included multiple CHK inhibitors and Aurora Kinase inhibitors, and warrant further investigations.

A novel finding of our study is the coupling of BET inhibition and MAPK activation and the intimate interaction between the two pathways. It is a common theme that complex networks of negative-feedback interactions exist in tumor cells and result in adaptive resistance upon targeted drug treatment [22]. For example, activated MAPK signaling induces the expression of many genes that paradoxically inhibit activation of the pathway. Of these, dual-specificity phosphatases (DUSPs) proteins dephosphorylate ERK [23], and Sprouty/Spred proteins act through suppressing the upstream activation of RAS by various receptor tyrosine kinases [15, 24, 25]. Accordingly, pharmacologic inhibition of MAPK signaling relieves MAPK-dependent feedback inhibition of DUSP and Sprouty/Spred proteins, followed by induction of RAS activation and rebound of MAPK pathway [26, 27]. Interestingly, we found that negative-feedback regulators of MAPK signaling including DUSP and Sprouty/Spred proteins were also transcriptionally controlled by BET bromodomain proteins, such that BET inhibition relieved this feedback, resulting in the hyperactivation of MAPK and an attenuation of the antitumor effects of BET bromodomain inhibitors. Therefore, the adaptive resistance to BET inhibitors can be, at least partially, overcome by co-administration of MEK inhibitors.

In summary, we revealed MAPK pathway activation as a cell-protective feedback mechanism that is triggered by BET bromodomain inhibition in ovarian cancer cells. Disruption of this feedback loop rendered cells susceptible to apoptosis and sensitized tumors to BET inhibitors. We envision that these discoveries will be generalizable to other solid and liquid tumor malignancies. Considering that both BET and MEK inhibitors are currently being actively evaluated for the treatment of various cancers as single agents in different stages of clinical trials (www.clinicaltrials.gov), our findings advocate a therapeutic strategy to potentially improve the efficacy by combining both agents that can disrupt the anticipated feedback activation in these tumors.

## MATERIALS AND METHODS

### Cell culture and reagents

Tumor cell lines were obtained from ATCC and were cultured in RPMI-1640 (Invitrogen) supplemented with 10% fetal bovine serum (Millipore). JQ1 and (−)-JQ1 were purchased from Millipore. I-BET151 and inhibitors library were purchased from Selleck Chemicals. All inhibitors were reconstituted in DMSO (Sigma-Aldrich) at a stock concentration of 10 mM.

### High-throughput small-molecule inhibitor screening

Drug screening was performed in a 96-well format. Cells were seeded at optimal density and treated with the indicated inhibitors at the same concentration (500 nM). Fresh medium and drugs were changed every three days. After six days of drug exposure, cells were imaged and viability was calculated using ArrayScan Infinity (Thermo Scientific) according to the manufacturer's instructions.

### Western blot

Cells were lysed in RIPA buffer (Tris pH 7.4 50mM, NaCl 150mM, NP-40 1%, SDS 0.1%, EDTA 2μM) containing proteinase inhibitors (Roche) and phosphatase inhibitors (Roche). The cell lysates (20μg protein) were subjected to SDS-PAGE and transferred to polyvinylidenedifluoride membranes. The membranes were blocked with 5% non-fat milk in TBST and incubated with specific primary antibodies. Detection was performed using SuperSignal WestPico chemiluminescent substrate (Thermo Scientific) followed by exposure to X-ray film. Antibodies against the following proteins were used: pAKT, pERK, pS6, Rab11, EGR1, cleaved PARP, cleaved Caspase3, BIM, pBAD and actin (Cell Signaling Technology).

### Microarray analysis and quantitative PCR

RNA was prepared with RNeasy plus mini kit (Qiagen) according to the manufacturer's protocol. Total RNA was subjected to microarray analysis using Affymetrix human genome U133 Plus 2.0. Three biological replicates per treatment group were included for statistical analyses. Affymetrix microarray probe-level data were normalized by Robust Multi-array Average (RMA) procedure. Differential gene expression was analyzed with linear models for microarray data (Limma). TaqMan gene expression assays (Applied Biosystems) were performed to verify the microarray results. Relative expression levels of each gene were normalized to human beta-actin. At least three biological replicates were included for each condition.

### Cell proliferation and apoptosis assays

Cell proliferation detection was performed in a 6-well format. Cells were seeded at optimal density and treated with the indicated inhibitors. Fresh medium and drugs were changed every three days. After two weeks of drug exposure, cell was fixed by methanol, stained by crystal violet and photographed by a digital scanner. Cell apoptosis was performed in a 96-well format. Cells were seeded at optimal density and treated with the indicated inhibitors. After incubation for 48 hours, cell apoptosis was detected by Caspase-Glo 3/7 assay kit (Promega) according to the manufacturer's instructions.

### *In vivo* study

Tumor cells (1×10^6^) were mixed with Matrigel (BD Biosciences) and subcutaneously implanted in the dorsal flank of BALB/c Nude mice. When tumor sizes reached approximately 200 mm^3^, mice were randomized into 4 groups of 6 mice each. One group of mice was treated with vehicle control (0.5% methylcellulose and 0.2% Tween-80), and the other three groups were treated with JQ1 (50 mg/kg/day), Trametinib (1 mg/kg/day) or JQ1 combined with Trametinib, respectively. Tumor volumes (6 animals per group) were measured with digital caliper and calculated as length×width^2^×0.52. The animals were housed in a specific pathogen free (SPF) animal facility in accordance with the Guide for Care and Use of Laboratory Animals and the regulations of the Institutional Animal Care and Use Committee.

### Cell cycle and apoptosis analysis

Cell cycle analysis was performed 24 hours after drug treatment. Cells were fixed in cold ethanol, resuspended in Propidium Iodide (PI)/RNase Staining Solution (Cell Signaling Technology) and incubated for 15 minutes at room temperature in the dark. For apoptosis analysis,cells were digested and collected with trypsin without EDTA, washed with PBS, incubated with Annexin V-FITC (Life Technologies) in room temperature for 15 minutes in dark and then incubated with PI for another 5 minutes. Flow cytometric analysis was performed on a FACS AriaII cytometer (BD Biosciences). Flow cytometry data was analyzed by using FlowJo software and the cell cycle was plotted as histogram after excluding doublets.

### Statistical analysis

In all experiments, comparisons between two groups were based on two-sided Student's *t*-test and one-way analysis of variance (ANOVA) was used to test for differences among more groups. *P*-values of <0.05 were considered statistically significant.

## SUPPLEMENTARY MATERIAL FIGURES AND TABLES


