# Supplementary material for: Concomitant BET and MAPK blockade for effective treatment of ovarian cancer
Source: Oncotarget. 2015 Nov 12;7(3):2545–54. doi: 10.18632/oncotarget.6309 (PMC4823054; doi:10.18632/oncotarget.6309)
Supplement: Supplementary file 1 [file oncotarget-07-2545-s001.pdf]

Concomitant BET and MAPK blockade for effective treatment of ovarian cancer

Supplementary Material

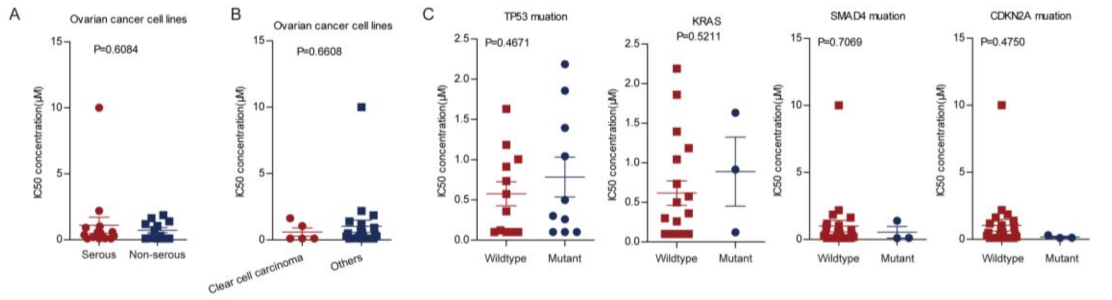

Supplementary Figure 1: JQ1 sensitivity did not correlated with histotype and mutation signature of ovarian cancer cells.

(A) Analysis of IC50 of JQ1 in serous and non-serous ovarian cancer cell lines. Student's unpaired *t*-test. (B) Analysis of IC50 of JQ1 in clear cell carcinoma and other ovarian cancer cell lines. Student's unpaired *t*-test. (C) Analysis of IC50 of JQ1 in TP53, KRAS, SMAD4 and CDKN2A wildtype and mutant ovarian cancer cell lines. Student's unpaired *t*-test.

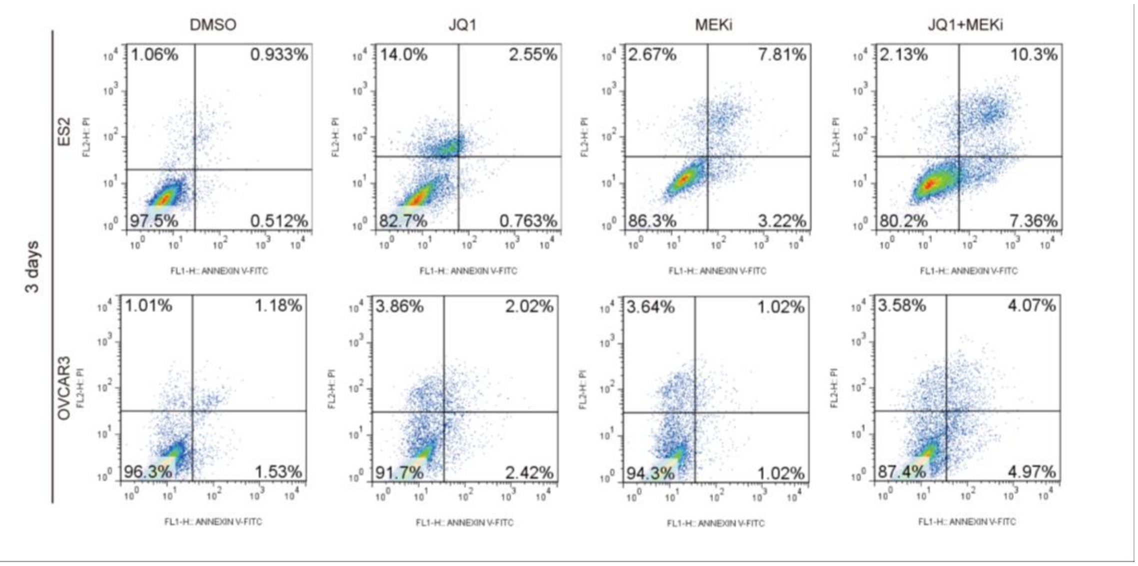

Supplementary Figure 2: Combination of BET and MEK inhibitors slightly induced ovarian cancer cell apoptosis in 3 days.

Cell apoptosis analysis following 3 days treatment with JQ1, MEKi (Trametinib), and JQ1+MEKi (Trametinib). Cells were dyed with Annexin V-FITC/PI.

Supplementary Table 1. IC50 of JQ1 for ovarian cell lines

Cell line	IC50 of JQ1
DOV13	0.594
HEY	1.006
COV318	0.2589
COV413B	0.192
COV504	0.4999
FU-OV-1	0.1
OAW28	0.1
OAW42	0.3602
OV7	0.5742
OV56	0.9134
OV90	0.1
OVCA420	0.1126
OVCA429	10
OVCA432	0.2742
OVSAHO	2.187
PE01	0.298
ES-2	1.043
OVISE	0.1
OVTOKO	0.1
RMG-1	0.1
TOV-21G	1.63
COV644	0.7339
EFO-27	0.3002
MCAS	0.1213
RMUG-S	0.1
COV362	1.858
IGROV-1	1.395
TOV112D	1.185

Supplementary Table 2. List of 180 drugs in screen

Item Name	Target
ABT-263 (Navitoclax)	Bcl-2
Obatoclax mesylate (GX15-070)	Bcl-2
TW-37	Bcl-2
ABT-199 (GDC-0199)	Bcl-2
Afatinib (BIBW2992)	EGFR
Gefitinib (Iressa)	EGFR
Lapatinib	EGFR
Neratinib (HKI-272)	EGFR
AZD9291	EGFR
Axitinib	VEGFR
Sorafenib (Nexavar)	VEGFR
Sunitinib Malate (Sutent)	VEGFR
Vandetanib (Zactima)	VEGFR
Dasatinib (BMS-354825)	Bcr-Abl
Nilotinib (AMN-107)	Bcr-Abl
Imatinib (Gleevec)	Bcr-Abl
Bosutinib (SKI-606)	Bcr-Abl
ABT-888 (Veliparib)	PARP
Olaparib (AZD2281)	PARP
Iniparib (BSI-201)	PARP
Rucaparib (AG-014699 , PF-01367338)	PARP
Bortezomib (Velcade)	Proteasome
MLN2238	Proteasome
INK 128 (MLN0128)	mTOR
AZD8055	mTOR
Deforolimus (Ridaforolimus)	mTOR
Temsirolimus (Torisel)	mTOR
Everolimus (RAD001)	mTOR
PD0325901	MEK
GSK1120212 (Trametinib)	MEK
AZD6244 (Selumetinib)	MEK
Binimetinib (MEK162, ARRY-162, ARRY-438162)	MEK
BEZ235 (NVP-BEZ235)	PI3K/mTOR
Apitolisib (GDC-0980, RG7422)	PI3K/mTOR
BKM120 (NVP-BKM120)	PI3K

CAL-101 (Idelalisib, GS-1101)	p110δ
GDC-0941	PI3K
Dovitinib (TKI-258)	c-Kit
BIBF1120 (Vargatef)	FGFR
Tandutinib (MLN518)	FLT3
Crizotinib (PF-02341066)	ALK
Alectinib (CH5424802)	ALK
Ceritinib (LDK378)	ALK
PHA-665752	c-Met
BMS 794833	c-Met
Vismodegib (GDC-0449)	Hedgehog
LDE225 (NVP-LDE225, Erismodegib)	Smoothened
MK-2206 2HCl	Akt
Ipatasertib (GDC-0068)	Akt
AZD5363	Akt
CHIR-99021 (CT99021) HCl	GSK-3
AZD1080	GSK-3
GSK2334470	PDK1
BX-795	PDK1
A-769662	AMPK
Phenformin HCl	AMPK
Ruxolitinib (INCB018424)	JAK
Tofacitinib (CP-690550) Citrate	JAK
Baricitinib (LY3009104, INCB028050)	JAK
Crenolanib (CP-868596)	PDGFR
Pazopanib	c-Kit
Masitinib (AB1010)	c-Kit
Regorafenib (BAY 73-4506)	c-Kit
Linsitinib (OSI-906)	IGF-1R
GSK1904529A	IGF-1R
NVP-AEW541	IGF-1R
BGJ398 (NVP-BGJ398)	FGFR
AZD4547	FGFR
Vemurafenib (PLX4032)	Raf
GDC-0879	Raf
Dabrafenib (GSK2118436)	Raf
Cabozantinib (XL184, BMS-907351)	Axl

R428 (BGB324)	Axl
BMS 777607	Axl
PF-562271	FAK
TAE226 (NVP-TAE226)	FAK
Birinapant	IAP
YM155	IAP
GSK2656157	PERK
SB 203580	p38 MAPK
BIRB 796 (Doramapimod)	p38 MAPK
Losmapimod	p38 MAPK
SP600125	JNK
JNK-IN-8	JNK
GDC-0994	ERK
SCH772984	ERK
Sotrastaurin (AEB071)	PKC
Enzastaurin (LY317615)	PKC
RKI-1447	ROCK
GSK429286A	ROCK
MK-1775	Wee1
LY2157299	TGF-beta/Smad
LDN193189	TGF-beta/Smad
LY2109761	TGF-beta/Smad
K02288	TGF-beta/Smad
Cisplatin	DNA/RNA Synthesis
Gemcitabine (Gemzar)	DNA/RNA Synthesis
Carboplatin	DNA/RNA Synthesis
Oxaliplatin (Eloxatin)	DNA/RNA Synthesis
Pemetrexed (Alimta)	DHFR
Adrucil (Fluorouracil)	DNA/RNA Synthesis
Capecitabine (Xeloda)	DNA/RNA Synthesis
Raltitrexed (Tomudex)	DNA/RNA Synthesis
EX 527	Sirtuin
Doxorubicin (Adriamycin)	Topoisomerase
Irinotecan	Topoisomerase
Epirubicin HCl	Topoisomerase
Topotecan HCl	Topoisomerase
BIBR 1532	Telomerase

WP1130	DUB
Mifepristone (Mifeprex)	Estrogen/progestogen Receptor
Toremifene Citrate (Fareston, Acapodene)	Estrogen/progestogen Receptor
Tamoxifen Citrate (Nolvadex)	Estrogen/progestogen Receptor
Fulvestrant (Faslodex)	Estrogen/progestogen Receptor
MDV3100 (Enzalutamide)	Androgen Receptor
Bicalutamide (Casodex)	Androgen Receptor
Flutamide (Eulexin)	Androgen Receptor
Leucovorin Calcium	
Abiraterone (CB-7598)	Androgen Receptor
Anastrozole	Aromatase
Letrozole	Aromatase
Exemestane	Aromatase
Abitrexate (Methotrexate)	DHFR
Temozolomide	Autophagy
Metformin HCl	Autophagy
Altretamine (Hexalen)	
CX-4945 (Silmitasertib)	PKC
Bardoxolone Methyl	IKK
Ifosfamide	DNA/RNA Synthesis
Tipifarnib (Zarnestra)	Transferase
BAY 11-7082 (BAY 11-7821)	IKK
BMS-345541	IKK
Celecoxib	COX
KU-60019	ATM
VE-822	ATR
Torin 2	ATM/ATR
VX-680 (MK-0457, Tozasertib)	Aurora Kinase
MLN8237 (Alisertib)	Aurora Kinase
Barasertib (AZD1152-HQPA)	Aurora Kinase
BAY 87-2243	HIF
SGI-1776 free base	Pim
AZD1208	Pim
Trichostatin A (TSA)	HDAC
Vorinostat (SAHA)	HDAC
Entinostat (MS-275, SNDX-275)	HDAC
Panobinostat (LBH589)	HDAC

Valproic acid sodium salt (Sodium valproate)	HDAC
Romidepsin (FK228, Depsipeptide)	HDAC
R935788 (Fostamatinib disodium, R788 disodium)	Syk
GS-9973	Syk
PAC-1	Caspase
Z-VAD-FMK	Caspase
DAPT (GSI-IX)	γ-secretase
MK-0752	γ-secretase
JNJ 26854165 (Serdemetan)	E3 Ligase
Nutlin-3	E3 Ligase
17-AAG (Tanespimycin)	HSP (e.g. HSP90)
Ganetespib (STA-9090)	HSP (e.g. HSP90)
PF-04929113 (SNX-5422)	HSP (e.g. HSP90)
AT13387	HSP (e.g. HSP90)
Docetaxel (Taxotere)	Microtubule Associated
Paclitaxel (Taxol)	Microtubule Associated
ABT-751	Microtubule Associated
AT7519	CDK
Flavopiridol (Alvocidib) HCl	CDK
PD 0332991 (Palbociclib) HCl	CDK
Roscovitine (Seliciclib, CYC202)	CDK
LY2835219	CDK
Dinaciclib (SCH727965)	CDK
AZD7762	Chk
LY2603618	Chk
XAV-939	Wnt/beta-catenin
(+)-JQ1	BRD
I-BET-762	BRD
EPZ-6438	EZH2
NU7441 (KU-57788)	DNA-PK
BI 2536	PLK
BI6727 (Volasertib)	PLK
Rigosertib (ON-01910)	PLK
PCI-32765 (Ibrutinib)	BTK

Supplementary Table 3. Analysis of protein phosphorylation in TCGA ovarian carcinoma

Protein	Residue	RPPA score (average)		p-value
		No BRD4 Amplification	BRD4 Amplification	
MAP2K1	pS217	0.07	-0.39	1.25E-04
AKT1/AKT2/AKT3	pS473	0.07	-0.35	2.39E-04
MAPK1/MAPK3	pT202	0.08	-0.43	3.76E-04
MAPK14	pT180	0.09	-0.42	4.73E-04
GSK3A/GSK3B	pS21	0.08	-0.4	5.95E-04
YAP1	pS127	0.07	-0.37	6.07E-04
AKT1/AKT2/AKT3	pT308	0.07	-0.33	8.06E-04
ARAF	pS299	0.07	-0.33	0.001
YBX1	pS102	0.06	-0.3	0.006
SRC	pY527	0.07	-0.31	0.011
RPS6	pS235	0.05	-0.26	0.019
NFKB1	pS536	0.05	-0.22	0.032
RPS6KA1	pT359	0.05	-0.24	0.036
RPS6	pS240	0.05	-0.23	0.039
